# Tolerable Upper Intake Level for Individual Amino Acids in Humans: A Narrative Review of Recent Clinical Studies

**DOI:** 10.1016/j.advnut.2023.04.004

**Published:** 2023-04-14

**Authors:** Rajavel Elango

**Affiliations:** 1 Department of Pediatrics, University of British Columbia, British Columbia, Canada; 2 British Columbia Children’s Hospital Research Institute, British Columbia Children’s Hospital, Vancouver, British Columbia, Canada; 3 School of Population and Public Health, University of British Columbia, British Columbia, Canada

**Keywords:** tolerable upper intake level, amino acids, humans, leucine, methionine

## Abstract

Individual amino acids are widely popular as supplements because of various perceived and real health benefits. However, currently, there are no recommendations set by national health agencies for tolerable upper intake levels (UL) for amino acids because of a lack of well-conducted human dose-response trials. In the past decade, under the initiative of the International Council on Amino Acid Science, a nonprofit organization, a series of UL human clinical studies were conducted. The goal of this narrative review is to summarize the studies on 6 essential amino acids (leucine, tryptophan, methionine, lysine, histidine, and phenylalanine), 2 nonessential amino acids (arginine and serine), and 2 nonproteinogenic amino acids (ornithine and citrulline) and provide the first set of ULs. A brief background of the concept of the DRI framework of UL, the concept of UL for amino acids, and a perspective of the results are also provided. The data suggest that in relatively healthy adult individuals, the tested amino acids are well tolerated, and ULs, or the no-observed-adverse-effect-level (NOAEL), lowest-observed-adverse-effect-level (LOAEL), can be determined. The ULs were for leucine—young (35 g/d), tryptophan (4.5 g/d), and leucine—elderly (30 g/d); NOAEL and LOAEL for methionine at 3.2 and 6.4 g/d, respectively; NOAEL for arginine (30 g/d); NOAEL and LOAEL for lysine at 6 and 7.5 g/d, respectively; NOAEL and LOAEL for histidine at 8 and 12 g/d, respectively; and NOAEL for phenylalanine (12 g/d), serine (12 g/d), ornithine (12 g/d) and citrulline (24 g/d). This first set of human UL data are hoped to help national and international agencies set safety standards for supplemental amino acids.

**Statement of Significance**

Amino acids are popular as supplements in humans because of several perceived and real health benefits; currently, there is no tolerable upper intake level established because of a lack of data. This review summarizes the first set of dose-response human clinical trials conducted on 10 amino acids, which will help establish tolerable upper intake levels and provide safety for supplemental amino acids.

## Introduction

Amino acids are the building blocks of mammalian tissue protein. Human proteins are made of predominantly 21 amino acids (the list now includes selenocysteine) for which tRNAs exist [1,2]. Amino acid functions in the body to form proteins, hormones, enzymes, and as regulatory compounds [3,4]. Beyond the proteinaceous amino acids, several physiologically important amino acids such as ornithine, citrulline, taurine, and hydroxyproline play key functional roles. Humans obtain individual amino acids from various protein food sources [4]. Food sources vary significantly in the amount and composition of the amino acids [[5], [6]], thus regulating protein and amino acid metabolism in both acute and chronic conditions. Individual amino acids are also widely popular as supplements [7] and are consumed because of various perceived health benefits.

To ensure humans can obtain adequate protein and amino acid intakes to benefit health, the DRIs, set both minimum (RDA) and maximum [tolerable upper intake level, (UL)] recommendations to balance deficiency compared with toxicity [8]. The most recent DRIs for protein and amino acids were published in 2005 when the Institute of Medicine (currently the National Academy of Sciences, Engineering, Medicine) set the recommendations. The topic of minimum recommendations for amino acids has been actively researched and published since 2005 DRIs [[9], [10], [11]]. However, the issue of maximum intakes of amino acids (that is, UL) has been less systematically studied and published from a human perspective. With the initiative of the International Council on Amino Acid Science (ICAAS), a nonprofit organization with a mandate to set guidelines for the safety of amino acids, a recent series of UL studies were conducted, and the results were published [[12], [13], [14], [15], [16], [17], [18], [19], [20]]. This review aims to summarize these human clinical trials of UL for amino acids to help regulatory agencies and bodies set amino acid supplement safety standards.

## Background, Which Led to A Series of UL Studies

The current DRI recommendations for amino acid intake, as discussed above, were set in 2005, and during the meetings held, it became apparent that establishing ULs would be challenging. As stated by Young [21], this was primarily because of insufficient data on dose-response relationships with increasing amino acid intakes. To address the lack of data and create a knowledge base on the impact of increased amino acid intakes on physiological organ systems, creating a framework for establishing ULs, ICAAS organized a series of 10 Amino Acid Assessment Workshops (AAWs) from 2001 to 2019. All the sessions, except the first AAW, have been published as Supplements in the Journal of Nutrition, and the reader is recommended to access the vast literature gathered on amino acid nutrition and metabolism [[21], [22], [23], [24], [25], [26], [27], [28], [29]]. The first 7 AAWs focused on extensive discussions on overall concepts of amino acid metabolism, specific amino acids, and mechanisms of action, with additional topics on risk characterization of adverse effects and regulatory aspects to be considered with excess amino acid intake [[21], [22], [23], [24], [25]]. The eighth, ninth, and tenth AAWs discussed data from systematic dose-response human clinical trials to establish ULs for leucine, tryptophan, arginine, methionine, histidine, and lysine [[28], [29], [30]]. These study data and additional studies on phenylalanine, serine, ornithine, and citrulline safety are discussed below, following a brief description of the UL concept, as it is applied by the DRI, with the modifications necessary for derivation of UL for amino acids.

## Background of DRI UL Concept

**FIGURE 1:**
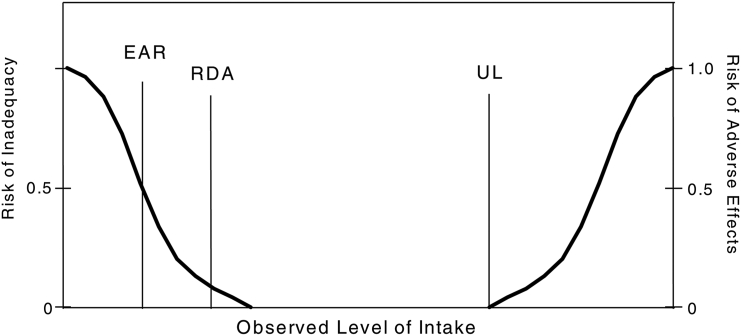
Dietary reference intakes paradigm of tolerable upper intake level (UL). EAR, estimated average requirement. Reproduced from reference [8] with permission.

The DRI framework for nutrients establishes recommendations for dietary intakes as an estimated average requirement (EAR), RDA to prevent deficiency (Table 1, Figure 1). EAR and RDA recommendations are a “minimum” to be considered adequate to meet 50% and 97.5% of the needs, respectively, in a population. It is important to remember that these values are fundamentally derived to prevent deficiency and do not necessarily refer to “optimal” needs in humans. Although EAR and RDA are determined experimentally, when data are not available to have an RDA, the AI is recommended based on observed intake data from a population [8]. With nutrients being consumed in excess of the body’s needs, there is a possibility of reaching intake levels where adverse effects can occur. The UL sets the experimentally determined safe upper limits, which is the “highest average daily nutrient intake level that is likely to pose no risk of adverse health effects to almost all individuals in the general population. As intake increases above the UL, the potential risk of adverse effects may increase” (Table 1) [8]. When data are available for an adverse event at a nutrient intake, it is suggested as the lowest-observed-adverse-effect-level (LOAEL), and at high intakes, if no adverse effects were observed, it is suggested as the no-observed-adverse-effect-level (NOAEL) [8].

**TABLE 1:** Definitions used to characterize nutrient intakes^1^

Term	Definition
Estimated average requirement (EAR)	the average daily nutrient intake level estimated to meet the requirement of half the healthy individuals in a particular life stage and gender group
Recommended dietary allowance (RDA)	the average daily dietary nutrient intake level sufficient to meet the nutrient requirement of nearly all (97% to 98%) healthy individuals in a particular life stage and gender group
Adequate Intake (AI)	the recommended average daily intake level based on observed or experimentally determined approximations or estimates of nutrient intake by a group (or groups) of apparently healthy people that are assumed to be adequate—used when an RDA cannot be determined
Tolerable upper intake level (UL)	the highest average daily nutrient intake level that is likely to pose no risk of adverse health effects to almost all individuals in the general population. As intake increases above the UL, the potential risk of adverse effects may increase
No-observed-adverse-effect-level (NOAEL)	highest intake (or experimental oral dose) of a nutrient at which no adverse effects have been observed
Lowest-observed-adverse-effect level (LOAEL)	the lowest intake (or experimental oral dose) at which an adverse effect has been identified

1 Definitions are from the National Academy of Medicine (formerly Institute of Medicine) (2005) [8].

Since the last DRI series of initiatives, there have been recent discussions to reconsider the DRI framework with a focus on chronic disease endpoints [31,32]. Because several nutrients are implicated in chronic diseases, the diseases might render nutrients deficient or, in some cases to be in excess to individuals [33]. Although these endeavors must be continued and pursued to ensure DRI guidelines for nutrient intakes remain relevant for promoting public health, for the purposes of setting the UL for amino acids, the below-described framework might be more relevant.

## UL Concept As Applied to Amino Acids

**FIGURE 2:**
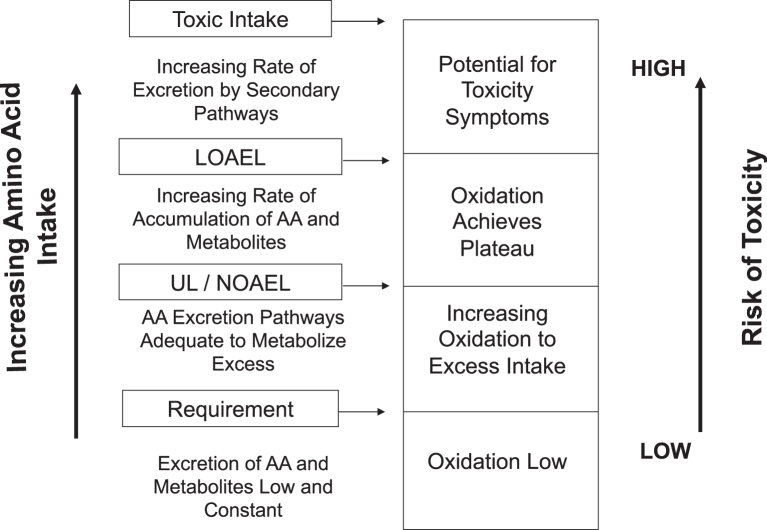
Conceptual description of the impact of increasing amino acid intakes and risk of toxicity. With amino acid intakes above the requirement, amino acid oxidative pathways continue to dispose of the additional amino acids. With intakes above the UL, oxidative pathways are saturated, and metabolites from amino acid catabolic pathways start to accumulate and increase risk of adverse events. AA, amino acid; LOAEL, lowest-observed-adverse-effect-level; NOAEL, no-observed-adverse-effect-level; UL, tolerable upper intake level. Adapted from reference [42] with permission.

It has been argued by Hayashi [34] and Loï and Cynober [35] that the ULs for nutrients need to have different risk characterization because they are different from food additives and contaminants [36], because nutrients nourish the body, obtained primarily from food, and are necessary to maintain health. Specifically with macronutrients, and thus amino acids, the conundrum exists because amino acids above normal consumption amounts are considered beneficial in some situations, such as exercise performance, recovery from injury, etc. [[37], [38], [39]]. It is also well known that toxic intake levels can be quite dangerous, as exemplified by the incidence of a potential methionine loading test dose of 96 g instead of 8.39 g [40]. Thus, there remains a rationale for ensuring ULs for amino acids be set. The criteria for setting ULs for amino acids were initially stated by Young [21], which arose from the discussions during the AAWs. It was stated that reliable, functional biomarkers of excess amino acids should be created, which included an accessible biomarker that can be sampled, measured reliably and objectively, and related to a physiological outcome/endpoint for that amino acid. It was also discussed by Bier [41] that amino acids labeled with stable isotopes could be used to develop pharmacokinetics with excess intakes of amino acids. We [42] extrapolated this to be applied to a framework (Figure 2) where we hypothesized that the oxidation of amino acids (measured using ^13^C-labeled amino acid tracers) would remain low and stable below the requirement. With intakes above the requirement, oxidation will increase to dispose of the excess amino acids until a “metabolic limit” is reached, after which oxidation will plateau. This “metabolic limit” identifies the UL and could also be the NOAEL (Figure 2). With intakes above the UL, risk of toxicity increases because the accumulation of amino acid metabolites increases, and an adverse event could be triggered, represented by the LOAEL (Figure 2). It was recognized that this framework might not apply to all amino acids, especially when metabolic pathways are more complex [42].

Taking the above concepts into account, the key to characterizing UL for amino acids in humans, systematic dose-response studies focusing on select biomarkers and overall adverse event reporting will be necessary. At present, studies on dose response with excess amino acids are available for 10 amino acids, which are discussed later in this review. To understand the results of such studies, it is also imperative to be aware of the usual daily intake of amino acids, which is described next.

## Amino Acid Intakes and Range of Exposures

Daily amino acid intakes are highly variable and influenced by several ethnic and socio-economic conditions. Because this manuscript is primarily focused on developed countries, daily intake of amino acids was obtained from the European Prospective Investigation into Cancer and Nutrition cohort [43] and the NHANES III 2005 in the United States [8] (Table 2). The European Prospective Investigation into Cancer and Nutrition cohort dietary data are collected using quantitative dietary questionnaires from 23 centers in 10 European countries: Denmark, France, Germany, Greece, Italy, the Netherlands, Norway, Spain, Sweden, and the United Kingdom. The data collected from a large samples size of 504,245 participants (147,259 male and 356,986 female) suggests that glutamic acid is the highest among all amino acids with a mean, 75th percentile of 13.6 and 16 g/d, respectively [43]. Among essential amino acids, leucine was the highest at the mean, 75th percentile of 5.6 and 6.6 g/d, respectively (Table 2). The NHANES III data are from a relatively smaller sample size of *n* = 1942–2533 participants but had similar data with glutamic acid being the highest at mean, 99th percentile of 19.6, 33.7 g/d, respectively; and leucine at mean, 99th percentile of 6.1, 14.9 g/d, respectively [8] (Table 2). Amino acid intakes parallel protein intakes, and in general, these studies reflect amino acid intakes primarily from food protein sources and are described here to provide context to the range of exposures normally seen in well-nourished individuals.

**TABLE 2:** Daily dietary intakes of amino acids^1^

Amino acid	EPIC 2022		NHANES/DRI 2005^2^	
	Mean	25th–75th percentile	Mean	99th percentile
g/d				
Alanine	3.2	2.4–3.9	5.3	8.5^4^
Arginine	3.6	2.8–4.3	4.9	10.1^4^
Aspartic acid	6.4	4.9–7.5	8.7	15.4^5^
Cystine/cysteine	0.8	0.6–1.0	1.2	2.2^4^
Glutamic acid	13.6	10.4–16.0	19.6	33.7^5^
Glycine	2.7	2.0–3.2	4.7	7.8^3^
Histidine	2.0	1.5–2.4	2.5	5.2^4^
Isoleucine	3.2	2.4–3.8	4.0	8.2^4^
Leucine	5.6	4.2–6.6	6.9	14.1^4^
Lysine	5.0	3.7–6.0	6.0	12.6^4^
Methionine	1.6	1.2–1.9	2.0	4.1^4^
Phenylalanine	3.2	2.4–3.8	4.4	7.7^5^
Proline	4.9	3.7–5.9	7.0	12.0^6^
Serine	3.3	2.6–4.0	4.5	7.9^5^
Threonine	2.8	2.1–3.3	3.5	7.1^4^
Tryptophan	0.8	0.6–1.0	1.0	2.1^4^
Tyrosine	2.6	1.9–3.1	3.6	6.4^5^
Valine	3.8	2.9–4.5	4.5	9.1^4^

Abbreviations: EPIC, European Prospective Investigation into Cancer and Nutrition.

1 Values are from the EPIC cohort (*n* = 504,245; 147,259 male and 356,986 female) [43].

2 Values are from NHANES III 2005 [8] (*n* = 1942–2533); ^3 m^ales, 19–30 y; ^4 m^ales, 51–70 y; ^5 m^ales, 31–50 y; ^6 m^ales, 14–18 y.

## Current Status of Knowledge

### Data available to set ULs for amino acids in humans

Based on the UL concept for amino acids elaborated earlier, systematic dose-response studies in humans were sponsored by ICAAS from 2012 to 2022. These series of studies are outlined in chronological order for leucine [12], tryptophan [13], leucine in the elderly [14], methionine [15], arginine [16], lysine [17], histidine [18], phenylalanine and serine [19], and ornithine and citrulline [20] in Table 3, with a brief description below. Several of the studies included subchronic intakes (defined as 4 wk of test amino acid intakes) and had participants in a repeated measures study design. Subacute was defined as <4 wk and acute was defined as <1 d, and these are different compared to food contaminants and additives [36].

**TABLE 3:** Summary of studies in humans for determining tolerable upper intake level of amino acids

Reference	Amino acid	Study design	Participants	Amino acid doses	Length of exposure	Primary outcomes	Other measures
Elango et al., 2012 [12]	Leucine	Acute, repeated measures study	*n* = 5 male; 27.2 ± 2.1 y; 23.7 ± 1.2 kg/m^2^	3.5, 10.5, 17.5, 35, 52.5, 70, and 87.5 g/d	8 h	Oxidation of 1-^13^C-Leucine, as a marker of metabolic excess	Blood ammonia, complete blood, and urine biochemistry
Hirasutka et al., 2013 [13]	Tryptophan	Subacute, randomly assigned, double-blind, placebo-controlled, crossover intervention study	*n* = 17 female; 20.2 ± 0.6 y; 20.1 ± 0.3 kg/m^2^	0, 1, 2, 3, 4, and 5 g/d	21 d	Surrogate biomarkers of tryptophan metabolites, including 3-hydroxykynurenine (3-HK) in blood and urine	Food intake, body weight, and general blood and urine biochemistry
Rasmussen et al., 2016 [14]	Leucine	Acute, repeated measures study	*n* = 6 male; 72.2 ± 3.5 y; 26.6 ± 5.0 kg/m^2^	10.5, 17.5, 24, 5, 31.5, 38.5, 45.5, and 52.5 g/d	8 h	Oxidation of 1-^13^C-Leucine, as a marker of metabolic excess	Blood ammonia, complete blood, and urine biochemistry, including amino acids
Deutz et al., 2017 [15]	Methionine	Subchronic, repeated measures study	n = 15 (9 male:6 female); 58.9 ± 6.1 y; 28.6 ± 0.9 kg/m^2^	0.6, 1.6, 3.2, and 6.4 g/d	4 wk	Plasma homocysteine concentrations	Health questionnaires, neurocognitive tests, complete blood and urine biochemistry, and bone mineral density
McNeal et al., 2018 [16]	Arginine	Subchronic, cross-sectional study	*n* = 74 (36 male:38 female); 22–57 y; ∼36 kg/m^2^	0, 15, and 30 g/d	90 d	Cardiovascular function (systolic and diastolic blood pressures)	Complete blood and urine biochemistry to test renal, metabolic function, and arginine metabolites
Hayamizu et al., 2019 [17]	Lysine	Systematic review	Included studies had *n* = 3–211 and age range, 1.6–76.7 y	0.2–17.5 g/d	1–1095 d	Report adverse events, including gastrointestinal tract symptoms such as nausea, stomachache, and diarrhea	—
Gheller et al., 2020 [18]	Histidine	Subchronic, repeated measures and cross-sectional study	*n* = 40 (*n* = 20 male, *n* = 20 female); ∼33 y; ∼24 kg/m^2^	4, 8, 12, and 16 g/d	4 wk	Complete blood biochemistry, including ferritin concentrations and liver enzyme values	Anthropometry, body composition, blood histamine concentrations, and sleep quality
Miura et al., 2021 [19]	Phenylalanine	Subchronic, repeated measures study	*n* = 23 male; ∼38 y; ∼23.5 kg/m^2^	3, 6, 9, and 12 g/d	4 wk	Complete blood biochemistry	Adverse event reporting, sleep quality, and body weight changes
Miura et al., 2021 [19]	Serine	Subchronic, repeated measures study	*n* = 30 male; ∼38 y; ∼23.5 kg/m^2^	3, 6, 9, and 12 g/d	4 wk	Complete blood biochemistry	Adverse event reporting, sleep quality, and body weight changes
Miura et al., 2022 [20]	Ornithine	Subchronic, repeated measures study	*n* = 23 male; ∼41 y, ∼23.8 kg/m^2^	3.2, 6, 9.2, and 12 g/d	4 wk	Complete blood biochemistry	Sleep quality, mental fatigue, and dietary intake surveys
Miura et al., 2022 [20]	Citrulline	Subchronic, repeated measures study	*n* = 23 male; ∼42 y, ∼23.6 kg/m^2^	6, 12, 18, and 24 g/d	4 wk	Complete blood biochemistry	Sleep quality, mental fatigue, and dietary intake surveys

### Leucine

Leucine is among the most popular amino acid supplement, together with isoleucine and valine, which forms the BCAA. Whey protein is enriched with leucine and has been promoted to help age-related muscle loss [38] and enhance performance during sports [37]. Using the concept of amino acid oxidation as an endpoint for the upper “metabolic limit” to dispose of excess amino acids (Figure 2) [42,44], Elango et al. [12] used L-1-^13^C-Leucine oxidation to ^13^CO_2_ as the primary endpoint with excess leucine intake. This was an acute (8 h) repeated measures study, where young men received 50, 150, 250, 500, 750, 1000, and 1250 mg/kg/d, corresponding to 3.5, 10.5, 17.5, 35, 52.5, 70, and 87.5 g/d for a 70 kg individual (Table 3). An upper limit to oxidize leucine was observed at 550 mg/kg/d, with elevated blood ammonia concentrations above normal beyond 500 mg/kg/d. Several blood and urine biochemistry variables, including plasma BCAAs, were measured, although no significant changes were observed. BCAA antagonism, where increased intakes of leucine reduced the plasma concentrations of isoleucine and valine, was observed. The UL was based on the maximum limit to oxidize leucine and blood ammonia increases and was set at 500 mg/kg/d, corresponding to 35 g/d for a 70 kg individual. To place the UL within context, the current adult leucine intake recommendations are kept at an EAR and RDA of 2.4 and 2.9 g/d, respectively. And as stated earlier, among the essential amino acids, leucine intake in the well-nourished adult population is at a mean of 5.6–6.9 g/d (Table 2).

### Tryptophan

Tryptophan is popularly used as a supplement for improving sleep patterns or mood because of its metabolite, serotonin [45]. Hirasutka et al. [13] designed a randomly assigned, double-blind, placebo-controlled crossover intervention study in young women, with test tryptophan intakes of 0, 1, 2, 3, 4, and 5 g/d each for a period of 21 d (Table 3). The primary endpoint for UL was based on blood and urine metabolites of the tryptophan catabolic pathway, including kynurenine, anthranilic acid, kynurenic acid, 3-hydroxykynurenine (3-HK), 3-hydroxyanthranilic acid (3-HA), and quinolinic acid. Urinary excretion of 3-HK increased 16-fold at the 5 g/d intake, compared to a ∼2.5-fold increase in 3-HA. This suggests a potential exceeding of the enzymatic capacity of kynureninase, which converts 3-HK to 3-HA. No other changes in sleep patterns or any other parameter were observed because of any of the intakes. Based on the 3-HK biomarker, a UL was suggested at 4.5 g/d for tryptophan (Table 4). In comparison, the mean daily intake of tryptophan is 0.8–1.0 g/d (Table 2).

**TABLE 4:** Summary of evidence for tolerable upper intake levels for amino acids^1^

Amino acid	UL	NOAEL	LOAEL	EAR^2^	RDA^2^
g/d					
Leucine—young men	35	—	—	2.4	2.9
Tryptophan	4.5	—	—	0.3	0.4
Methionine^3^	—	3.2	6.4	1.1	1.3
Leucine—elderly men	30	—	—	2.4	2.9
Arginine^4^	—	30	—	—	—
Lysine	—	6	7.5	2.1	2.7
Histidine	—	8	12	0.8	1.2
Phenylalanine^3^	—	12	—	1.9	2.3
Serine	—	12	—	—	—
Ornithine	—	12	—	—	—
Citrulline	—	24	—	—	—

Abbreviations: EAR, estimated average requirement; LOAEL, lowest-observed-adverse-effect-level; NOAEL, no-observed-adverse-effect-level; UL, tolerable upper intake level.

1 Values are mean UL per day for amino acids from diet and supplements; NOAEL; LOAEL; Values are based on 70 kg individuals, aged >19 y.

2 Mean daily requirement for amino acids expressed as EAR and RDA (DRI 2005) [8].

3 Daily requirements are recommended as methionine + cysteine, and phenylalanine + tyrosine.

4 Studies included provision of amino acids as .HCl supplements.

### Leucine—elderly

Considering that leucine is a popular supplement to prevent age-related loss of muscle mass, the next study focused on elderly men aged >70 y [14] and used the same UL concept applied earlier in young men–oxidation of 1-^13^C-Leucine to ^13^CO_2_. Because the UL for young men was determined at ∼500 mg/kg/d, reducing the range of excess leucine intakes was considered safer. Leucine intakes of 150, 250, 350, 450, 550, 650, and 750 mg/kg/d corresponding to 10.5, 17.5, 24,5, 31.5, 38.5, 45.5, and 52.5 g/d for a 70 kg individual were tested in an acute-repeated measures study design (Table 3). An upper limit to oxidize leucine was observed at 430 mg/kg/d, which corresponds to a UL of 30 g/d (Table 4). This value is numerically lower than the 35 g/d determined in young men; however, it was not statistically different. Thus, there might be a slight age-related decline in the ability to dispose of excess leucine. The study also observed that leaner participants with altered body composition had a different blood ammonia response compared to the increase above normal observed with increasing leucine intake [46]. Clearly, body composition plays a role in the disposal of excess leucine intake and needs to be explored in the future.

### Methionine

Methionine is used in the clinical context as a methionine loading test to examine the elevation in homocysteine concentrations as a potential risk marker for older adults with atherosclerosis [15,47]. This is primarily because of the involvement of methionine in the transmethylation cycle to produce homocysteine as an intermediate before being either remethylated to methionine (remethylation cycle) or synthesized cysteine (transsulfuration pathway) [47,48]. Increased concentrations of methionine have been shown to be toxic; thus, Duetz et al. [15] designed a repeated measures study in ∼59 y old men and women, with intakes of 9.2, 22.5, 46.3, and 91 mg/kg/d, corresponding to 0.6, 1.6, 3.2, and 6.4 g/d for a 70 kg individual for 4 wk periods (Table 3). Plasma homocysteine concentrations were elevated at 6.4 g/d and were set as the LOAEL, with the intake of 3.2 g/d set as the NOAEL (Table 4). All other biochemical measures and tests of neurocognition and body composition remained unchanged. Current mean intakes for methionine are reported at 1.6–2 g/d (Table 2).

### Arginine

Arginine is the precursor for nitric oxide synthesis, a major vasodilator in the body [49]. Interest in arginine as a supplement because of perceived cardiovascular benefits [50] in overweight and at-risk individuals led McNeal et al. [16] to test in a cross-sectional design arginine intakes of 0, 15, and 30 g/d for 90 d (Table 3). The study population was both men and women with a BMI (in kg/m^2^) of ∼36. The primary endpoints were on cardiovascular function, including blood pressure, and additional blood biochemistry to test renal, hepatic, metabolic functions, and arginine metabolites in plasma. No significant changes with a specific pattern were observed with intakes of arginine, and the 30 g/d dose was well tolerated. The NOAEL for arginine was set at 30 g/d (Table 4), and the comparative mean intake in humans is 3.6–4.9 g/d (Table 2).

### Lysine

Lysine, usually the first limiting amino acid in cereal-based diets, has been studied extensively to ensure lysine adequacy can be ensured for populations, including fortification studies [51]. Thus, Hayamizu et al. [17] performed a systematic review of all studies published that had tested the dose-response effects of oral lysine consumption. Seventy-six studies were selected for evaluation, of which 12 were included in the systematic review. The included studies had variable sample sizes from *n* = 3 to *n* = 211 and had a wide range of age (∼2–77 y) and in the supplement lysine doses (0.2–17.5 g/d) (Table 3). The primary endpoints for lysine excess were reports of adverse events, including gastrointestinal insults, headache, and nausea. Based on the reported adverse impact of diarrhea, the LOAEL was set at 7.5 g/d and the NOAEL at 6 g/d (Table 4). The mean usual intake has been reported to be 5–6 g/d, and the reported 99th percentile intake of 12.1 g/d is clearly above the LOAEL (Table 2). However, it is also likely that the reasons for the gastrointestinal insults in studies with lysine supplements are possible because of the form of lysine (lysine.HCl), as opposed to the natural lysine in foods, which is likely from animal protein sources such as milk, beef etc., that are rich in lysine.

### Histidine

Histidine labeled an essential amino acid has been debated because of the fact that endogenous sources of histidine exist, such as carnosine and hemoglobin. Histidine has also been implicated with health benefits, including anti-inflammatory and antioxidant [52], and thus is consumed as a supplement. Gheller et al. [18] designed a 4-wk repeated and cross-sectional study in ∼40 young men and women with histidine intakes of 4, 8, 12, and 16 g/d (Table 3). The 12 g/d had reduced ferritin concentrations, and the 16 g/d dose had elevated liver enzymes. No other changes in body composition, sleep quality, or histamine concentrations were observed. However, because of the adverse effects observed at the 12 and 16 g/d, the 8 g/d was set as the NOAEL and 12 g/d as the LOAEL for histidine (Table 4). Current mean intakes are 2–2.5 g/d (Table 2).

### Phenylalanine and serine

Phenylalanine, an essential amino acid, is necessary for neurotransmitter synthesis via tyrosine [53]. Serine, a nonessential amino acid, is key for synthesizing other compounds, such as sphingolipids and amino acids, including glycine [54]. Miura et al. [19] conducted 2 clinical trials in young Japanese men with test doses of phenylalanine and serine at 3, 6, 9, and 12 g/d in a repeated measures design for 4 wk (Table 3). Complete blood and urine biochemistry, with adverse event reporting and measurement of sleep quality, was conducted. No adverse events correlated with any of the test intakes, and no remarkable changes in biochemistry variables were observed. Thus, the NOAEL for phenylalanine and serine was set at 12 g/d (Table 4).

### Ornithine and citrulline

Ornithine and citrulline are nonproteinogenic amino acids and are present naturally in food sources. However, both are popular as supplements because of the perceived benefits of ornithine as a sleep enhancer and improvements in liver function, and citrulline because of its performance-enhancing effects in high-intensity exercise [39]. Miura et al. [20] conducted a repeated measures study in young Japanese men, with ornithine doses at 3.2, 6, 9.2, and 12 g/d; and citrulline doses at 6, 12, 18, and 24 g/d for 4 wk (Table 3). Primary endpoints were complete blood biochemistry, measurements of sleep quality, mental fatigue, and dietary intake. No major changes were observed in any of the blood parameters, and adverse event reporting was not related to increased doses of either amino acid. Thus, the NOAEL was set at 12 g/d for ornithine and 24 g/d for citrulline (Table 4).

### Amino acids for which data are not available to set ULs

For the remaining amino acids, there have been no new systematic clinical studies conducted. Among the remaining indispensable amino acids, there are no studies available for isoleucine, valine, and threonine in healthy individuals. In patients with spastic paraparesis, supplements of 4–6 g threonine/d [55], and in patients with amyotrophic lateral sclerosis, isoleucine 8 g/d and valine 6.4 g/d were shown to be safe [56]. Based on the limited data, no UL recommendations for the remaining indispensable amino acids can be made at this point.

Similarly, there are no systematic dose-response clinical trials among the remaining dispensable amino acids. The most commonly discussed and reviewed amino acid has been glutamate [57], primarily because of the use of monosodium glutamate (MSG) as a flavoring agent in several cultures [58]. Glutamate is also the highest among all amino acids to be consumed by humans on a routine basis, primarily from food protein sources (Table 2). MSG consumption is relatively lower but highly variable, with ∼0.6 g/d in North America and Europe, to 1–3 g/d in Asian countries [58]. Although concerns were raised about MSG causing severe responses in some individuals with anecdotal reports of fluttering heartbeats, tingling sensation, nausea, etc., controlled experiments in humans at the normal consumption concentrations of MSG failed to show any adverse reports in humans [59]. Although a specific UL has not been established, the most recent reports suggest an admissible daily intake of 240 mg/kg/d, which equates to ∼17 g/d for a 70 kg adult combined for glutamate and glutamate salts [58,60].

## Perspectives

The current review summarized the first set of data collected thus far to recommend the UL for amino acids. This included 6 of the 9 essential amino acids (leucine, tryptophan, methionine, lysine, histidine, and phenylalanine), 2 nonessential amino acids (arginine and serine), and 2 nonproteinogenic amino acids (ornithine and citrulline). ULs have been recommended for all these amino acids under Table 4. This is a major advancement compared to the 2005 DRI report when the report concluded that “Since ULs could not be established for any of the amino acids (some of which are known to result in toxic effects at high doses) because of insufficient data on dose-response relationships, more data are needed on adverse effects of high intakes of amino acids.” The data presented here should allow regulatory agencies to start establishing the amino acid ULs. It should be stated that this review focused only on the recent human studies with a focus on amino acid ULs. There are several animal (primarily rodent) studies on amino acid UL, and these were recently reviewed by Blachier et al. [61]. Animal model studies to determine amino acid tolerability need the inclusion of uncertainty factors to scale the values to be translatable to humans; however, such factors are not universally agreed upon [58,61]. Readers are recommended to review this [61] and the earlier DRI report [8] for additional details. Furthermore, this review does not attempt to outline/characterize any health benefits, perceived or real, for any of the amino acids or recommend doses of supplements for consumption. The goal was to present the human data generated with high quality in study design and data collection on several endpoints on the impact of amino acid excess, which, as stated by DRI, has the greatest weight in setting human guidelines in the future.

An additional point of importance is the fact that in the studies described above, the amino acids tested as supplements in the studies were all in the L-form of the highest purity possible (99.9%), sourced from tested and reliable manufacturers. The ULs being recommended (Table 4) are for similar supplements, which is a key consideration that regulatory agencies will have to emphasize in future guidelines. Indeed, the quality of amino acid supplements has been discussed earlier by Oketch-Rabah et al. [62], Smriga [63], and Karakawa et al. [64].

Individual amino acid consumption in excess tends to cause amino acid imbalances in vivo and can have both immediate (for example, loss of appetite) and long-term impacts (for example, fatty liver) [65,66]. Amino acid antagonism has also been well described, especially by Harper [66], where amino acids compete for the same enzyme complex in the degradative pathway, as with the BCAAs (leucine, isoleucine, and valine) or the same transport system (lysine and arginine) [67]. In the leucine UL studies described above, leucine intakes above ∼17.5 g/d [12] and ∼10.5 g/d [14] in young men and elderly men, respectively, plasma amino acid concentrations of isoleucine and valine decreased significantly compared to normal consumption amount of ∼3.5 g leucine/d. This is because all 3 BCAA share a common catabolic pathway, with the enzyme branched-chain keto dehydrogenase (BCKDH) as the regulatory enzyme in the irreversible degradation of BCAA. It has been suggested earlier that leucine is the primary regulator of this enzyme, and thus high intakes of leucine stimulate increased activity of BCKDH [46], thus increasing the catabolism of isoleucine and valine. In young, growing animal models, BCAA antagonism has been shown to cause growth deficits [66], although it is not clear what the long-term impact of this effect is in adult humans. Lysine and arginine share the same cationic amino acid transport systems (primarily system y^+^L) for transport across cell membranes. In adult humans increasing intakes of arginine with a constant intake of lysine at normal amounts was shown to impact ^13^C-Lysine oxidation to ^13^CO_2_ and reduce plasma lysine concentrations below normal ranges [67]. The long-term health impacts of individual amino acid supplements in humans are not known and should be characterized to better understand amino acid imbalances and antagonism.

## Strengths and Limitations

There are a few limitations in this review to be borne in mind. The review describes studies in healthy individuals, mostly young adults, with some studies in the elderly - leucine [14], older adults - methionine [15], and 1 study in obese individuals –arginine [16]. The leucine, phenylalanine, serine, ornithine, and citrulline studies included only men [12,19,20]; the tryptophan study included only women [13]; and the lysine systematic review analysis included individuals of a wide range in age, and sex [17]. It is not known whether sex has an effect on the toxicity of different amino acids. Furthermore, these studies were mostly in adults in apparently good health. There is no evidence that other age groups, life stages, and individuals with the disease would have the same tolerable upper intake for amino acids. Thus, data generalizability can be difficult, and the UL values must be utilized with the data caveats in mind. The supplement form for lysine, arginine, and ornithine in the current set of studies was as .HCl, which improves amino acid stability and solubility. The impact of these forms in certain populations can be quite different from healthy individuals who can tolerate the chloride form of supplement. The length of exposure in these UL studies was relatively short (1–90 d); it is unknown if longer periods of supplement use will lead to amino acid metabolic pathways adapting in different ways, such that toxic effects might appear, or other catabolic pathways might be upregulated. Some of the strengths of the review is that the studies reviewed utilized a repeated measures design, with multiple test amino acid intake levels. Such dose-response studies with a range of intakes tested can reduce interindividual variability, provide a robust data set and increase the level of confidence in the recommended UL values. The summarized data were of high-quality amino acid supplements consumed by relatively healthy individuals consuming adequate calories and, in general, reflects a “real world” situation, and data can be interpreted with confidence to be applied for public health guidelines for adults.

In summary, amino acids are popular as supplements because of various reasons. The DRIs, which recommend ULs for the safety of nutrients, including amino acids, could not establish ULs for amino acids because of the lack of systematic dose-response human clinical trials. Under the initiative of the ICAAS, in the past decade, a series of human clinical studies were conducted. Based on this first set of data UL/NOAEL/LOAEL have been established for leucine, tryptophan, methionine, arginine, lysine, histidine, phenylalanine, serine, ornithine, and citrulline (Table 4). This is a major achievement that will allow DRIs to set safety limits on amino acid intakes and will also help regulatory agencies guide national quality standards for the supplement industry. It is also hoped that this summary provides researchers in the field of amino acid nutrition safety data, which will be useful in exploring other helpful benefits of amino acids during different pathologies and life conditions. Optimizing amino acids in the diet has potential implications for regulating health in humans.

## Funding

The author reported no funding received for this study.

## Author disclosures

RE has received travel and accommodation support to speak at meetings organized by the International Council on Amino Acid Science (ICAAS) and served on the Scientific Advisory Committee of ICAAS.

## Acknowledgments

The sole author was responsible for all aspects of this manuscript, including drafting and summarizing the data, and was primarily responsible for the final content.
